# Function of Adenosine 2A Receptor in High-Fat Diet-Induced Peripheral Neuropathy

**DOI:** 10.1155/2020/7856503

**Published:** 2020-05-22

**Authors:** Ji Li, Huan-Qiu Liu, Xin-Bai Li, Wen-Jun Yu, Tao Wang

**Affiliations:** ^1^Department of Anesthesiology, The First Hospital of Jilin University, Changchun, China; ^2^Department of Hand Surgery, The First Hospital of Jilin University, Changchun, China

## Abstract

Peripheral diabetic neuropathy (DPN) is a complication observed in up to half of all patients with type 2 diabetes. DPN has also been shown to be associated with obesity. High-fat diet (HFD) affects glucose metabolism, and the impaired glucose tolerance can lead to type 2 diabetes. There is evidence to suggest a role of adenosine 2A receptors (A2ARs) and semaphorin 3A (Sema3a) signaling in DPN. The link between the expression of Sema3a and A2AR in DPN was hypothesized, but the underlying mechanisms remain poorly understood. In this study, we investigated the regulation of Sema3a by A2AR in the spinal cord and the functional implications thereof in DPN. We examined the expression of A2ARs and Sema3a, as well as Neuropilin 1 and Plexin A, the coreceptors of Sema3a, in the dorsal horn of the lumbar spinal cord of an animal model with HFD-induced diabetes. Our results demonstrate that HFD dysregulates the A2AR-mediated Sema3a expression, with functional implications for the type 2 diabetes-induced peripheral neuropathy. These observations could stimulate clinical studies to improve our understanding on the subject.

## 1. Introduction

Peripheral diabetic neuropathy (DPN) is a common diabetes complication that affects approximately 50% of type 2 diabetes patients and brings an enormous strain on both patients and society [1]. While research showed that hyperglycemia plays a key role in DPN by causing systemic and neuronal oxidative stress [2–4], clinical trials have also shown that obese patients may show symptoms of peripheral neuropathy even if they have normal blood sugar level [5–7]. Likewise, studies in animal models of obesity have reported peripheral neuropathy in obese animals [8, 9]. However, very little is known about the neurobiological mechanisms linking obesity with peripheral neuropathy.

It is well established that high-fat diet (HFD) can affect glucose metabolism, and the impaired glucose tolerance can lead to the type 2 diabetes [10]. HFD may cause large myelinated nerve and small sensory nerve fiber damage, thus leading to peripheral neuropathy [8, 9, 11]. The study of HFD-fed C57BL/6 mice showed deficits in motor and sensory nerve conduction velocity (NCV), thermal hyperalgesia, and reduced mean dendrite length [11].

The mechanisms underlying these processes may involve the regulation of semaphorins. Semaphorins are a large family of membrane-associated and secreted proteins participating in multiple cellular processes. Semaphorins are bifunctional signaling molecules capable of growth promoting or growth inhibitory effects [12]. This diversity of functions is related to the formation of specific receptor complexes. Together with their receptors, the neuropilins and the plexins, semaphorins are the constituents of a complex regulatory system responsible for axon guidance during the development of the central nervous system [13, 14].

Sema3a, one of the members of semaphorin family, acts *in vivo* as a repulsive guidance cue for the peripheral projections of embryonic dorsal root ganglion (DRG) neurons. Sema3a binds with high affinity to Neuropilin 1 on growth cone filopodial tips. Although Neuropilin 1 is required for Sema3a action, it is incapable of transmitting a Sema3a signal to the growth cone interior. Instead, the Sema3a/Neuropilin 1 complex interacts with another transmembrane protein, plexin, on the surface of growth cones. The intracellular domain of plexin is responsible for initiating the signal transduction cascade which leads to growth cone collapse, axon repulsion, or growth cone turning [15].

In turn, Sema3a is regulated through other signaling pathways. It was demonstrated that expression of this protein can be modulated by stimulation of A2 adenosine receptors (A2ARs) [16]. The A2ARs were identified as significant regulators of HFD-induced hallmarks of type 2 diabetes. Administration of HFD for sixteen weeks was reported to vastly upregulate the expression of the A2bAR in control mice, while A2bAR knockout mice under this diet developed greater obesity and hallmarks of type 2 diabetes [17].

A2ARs were shown to be involved in the control of neuropathic pain caused by peripheral nerve injury and characterized by a significant decrease of the mechanical allodynia and a suppression of thermal hyperalgesia and allodynia [18]. Downregulation of adenosine A2A receptors was found to be relevant for the development of hypertensive diabetic nephropathy [19] and diabetic retinopathy [20].

Importantly, the possibility of upregulating A2ARs in DPN has been studied in humans. For example, BVT115959, an A2ARs agonist derived from a marine natural product [21], was shown to be effective in 3 × 7 mg oral daily dose in a clinical trial for diabetic neuropathic pain [22]. This particular clinical trial was ended because the company stopped its research on small molecules. However, the preliminary findings indicate that selective and potent A2AR agonists have high potential for DPN treatment. More recently, studies have shown that stimulation of A2ARs could increase the expression of Sema3a [16]. Previous studies have shown that Sema3a can attenuate hyperalgesia in the spinal cord of the nerve growth factor- (NGF-) induced neuropathic pain model [23]. On the basis of these findings, we hypothesized that A2ARs may play a critical role in the DPN development and progression via regulation of Sema3a in the spinal cord.

In the current work, we looked at the role of A2A receptor stimulation and Sema3a levels in the spinal cord in peripheral neuropathy using an animal model with HFD-induced diabetes. We also investigated the expression of A2ARs, Sema3a, Neuropilin 1, and Plexin A in the dorsal horn of the lumbar spinal cord. Finally, we studied the effects of repeated intraperitoneal administration of selective A2ARs agonist SCH58261 on the peripheral neuropathy in HFD-fed mice.

## 2. Materials and Methods

### 2.1. Animals

Male C57BL/6J mice were obtained from the Experimental Animal Center at Jilin University and were given access to food and water *ad libitum*. The study involving animals was approved by the Institutional Animal Care and Use Committee of the Second Hospital of Jilin University. All the guidelines under the “Guide for the Care and Use of Laboratory Animals” (Institute of Laboratory Animal Resources, Commission on Life Sciences 2011) were strictly followed.

### 2.2. High-Fat Diet Treatment

We used a protocol similar to the one in a previous study [24] to feed the mice weighing 23-25 g at eight weeks with normal mouse chow (SLACOM, Shanghai, China) and/or high fat diet (D12330 formula, 58 kcal percent fat with corn starch, Research Diets, In., New Brunswick, NJ)for 24 weeks. In a separate group of high-fat diet- (HFD-) treated mice, vehicle (10% DMSO, 40% PEG300, 5% Tween-80, and 45% saline) or SCH58261 treatment (1 or 10 mg/kg; IP) was administered once daily for one week starting at week 24.

### 2.3. Blood Glucose and Weight Monitor

Blood glucose monitoring and weight measurements were performed on a weekly basis (glucose diagnostic reagents; Kinbio Tech, Shanghai, China) [8, 25, 26]. Mice were fasted for 3 hours before the blood was collected from the tail. In our research, after 24 weeks of HFD, the mice had blood glucose of around 8.1-9.0 mmol/l. These findings were consistent with the prior research on prediabetes and obesity caused by high-fat diets.

### 2.4. Measurement of Nerve Conduction Velocity (NCV)

Animals were anesthetized with isoflurane using approved vaporizer and scavenging system. The flank was treated with betadine, and a 0.5 cm long incision in the flank was made to expose the sciatic nerve, followed by the separation of underlying musculature by blunt dissection. A thermistor probe was placed adjacent to the nerve, the wound was closed with a skin clamp, and a rectal probe was positioned. Rectal temperature was maintained at 37°C by a thermal pad. Nerve and ambient temperature was maintained using a heat lamp connected to a temperature regulator that automatically turned off the heat source when the thermistor probe detected temperatures above 37°C. The heating lamp was not allowed closer than 2 feet (60 cm) from the animals, and the animals had their head and abdomen covered with a heat reflective material. The nerves of animals were stimulated (200 mV, 50 *μ*s duration square wave stimulus every 2 s) using fine needle electrodes that were placed at the sciatic notch. The evoked electromyogram of interosseous muscles was recorded by two fine needle electrodes. Then, the thermistor probes were removed; the skin incision was closed with wound clips and coated with betadine, and the animals were withdrawn from anesthetic, followed by their monitoring in a temperature-controlled chamber. Upon waking, animals were observed for 5 min prior to their return to their regular cages. The procedure took less than 5 min and the skin wound was observed to heal within 1 week, allowing repeated measurements on alternate weeks during the course of each study.

### 2.5. Behavioral Tests

All tests were carried out on mice kept on either normal diets or HFD for 24 weeks. The mice were first acclimatized to a behavioral apparatus. During the test days, the mice were put into a behavioral apparatus for 30 min to acclimatize to the setting. Tactile reaction testing was done using the von Frey assay. Specifically, the mice were put on a wire mesh grid in a transparent plastic cage allowing access to the mice hind paws. In order to assess peripheral neuropathy, the mechanical withdrawal thresholds were then evaluated using the von Frey monofilament. The midplantar surface of every hind paw was perpendicular to the sequence of the von Frey filaments (0.02, 0.04, 0.07, 0.16, 0.5, 0.6, 1.0, and 1.4 g force) (Stoelting, Wood Dale, IL, USA). The filaments were buckled for approximately 2–3 s, with the intervals of approximately 5 min. The 0.16 g force von Frey filament was used to start the experiment. The positive reaction was described as a hind-power retreat from the stimulus. Whenever a positive stimulus response was given, the next lowest of the von Frey filaments were used, and the next higher filaments was applied when a negative response occurred. After the first reaction measurement, the test consisted of five further stimuli. In order to establish a 50% withdrawal threshold, the up-down approach was used [27, 28].

Following von Frey's experiment, the heat sensitivity of the mice was assessed by a hot plate test. Specifically, the animals were placed on a 55°C hot plate, and a video camera was used to record their behavior. The latency was calculated for licking the front of rear paws. The examiners blinded to the experimental circumstances performed all the calculations.

### 2.6. Western Blot

Animals were anesthetized with 4% isoflurane and decapitated rapidly. The entire lumbosacral spinal cord section was collected. The tissue was homogenized in the lysis buffer containing 50 mM Tris-HCl (pH 8.0), 1 mM EDTA, 150 mM NaCl, 0.5% Triton X-100, and a full protease inhibitor cocktail. The homogenized tissue samples were incubated on ice for 30 min. The samples were centrifuged at 15,000 rpm (Eppendorf 5415C, Shanghai, China) at 4°C and stored at -80°C. To determine the protein levels, the BCA Protein Assay Kit (Abcam, Shanghai, China) was used. Protein samples (50 *μ*g) were subsequently separated on 8% polyacrylamide gel (Tris-HCl) and transferred to a polyvinylidene difluoride membrane (Millipore, Billerica, MA, USA) at room temperature for 1 h at 110 V. The membrane was incubated with the primary antibody at 4°C overnight. The primary antibodies that we used included anti-semaphorin 3A antibody, anti-Neuropilin 1 antibody, anti-adenosine receptor A2A antibody, and anti-Plexin A1 antibody (Abcam, Shanghai, China). After washing, the membranes was incubated with HRP- (horseradish peroxidase-) linked secondary antibody (GE Healthcare, Piscataway, NJ, USA) at room temperature for 2 h. The luminescent signals were generated using the electrochemiluminescent kit (GE Healthcare, Piscataway, NJ, USA) and detected by Kodak X-ray footage exposure (X-OMAT; Kodak, Shanghai, China). The membrane was washed to reprobe for anti-*β*-actin antibody (1 : 1000, Santa Cruz, Shanghai, China) using the stripping buffer. The NIH ImageJ software was used to quantify the protein levels by densitometry.

### 2.7. Intraepidermal Nerve Fiber Density Quantification

After the skin tissue has been kept for 18 to 24 h in Zamboni's fixative and cryoprotected in 20% sucrose overnight, it was embedded in OCT (optimal cutting temperature) compound and cut into 20 *μ*m (mice footpad) cryostat sections (Leica Microsystems CM 1850, Germany). Three sections of each tissue were randomly chosen and immunostained with rabbit anti-protein 9.5 (PGP 9.5). PGP 9.5-positive fibers originating from and crossing the dermal-epidermal junction of the skin were quantified under a light microscope in three fields of each segment at magnification of ×200. Within the epidermis, secondary branches and fragments were not counted. The section length was evaluated, and the linear epidermal innervation density was calculated as intraepidermal nerve fiber density (IENFD) (expressed as IENF/mm) [29].

### 2.8. Statistical Analysis

GraphPad Prism 5.0 (GraphPad Software, San Diego, CA, USA) was used for all statistical analyses. All quantitative data were provided as the means ± SEM. The *t*-test was used to analyze the behavioral tests. Variance analysis (ANOVA) was used after the administration of pharmaceuticals to assess the behavioral data. Multiple comparisons were performed following a main ANOVA effect using Tukey's post hoc test. Significance was set at *p* < 0.05.

## 3. Results

### 3.1. Effects of HFD Treatment on Body Weight, Blood Glucose Level, and Food Intake

Body weight, blood glucose, and dietary intake of mice on normal diet and HFD were monitored (Table 1). In general, gains in body weight were observed over time. After the first week of HFD, the mice showed a slight increase in body weight and food intake, compared to the normal-diet-fed mice, but showed no changes in blood glucose. The HFD mice had greater weight gains relative to the normal diet mice after 16 or 24 weeks. The average one-day food consumption of mice on HFD in comparison with mice on normal diet has also been considerably greater. In addition, the blood glucose levels in the HFD-fed mice were higher than in the normal diet mice at 16 and 24 weeks of HFD.

### 3.2. HFD Treatment-Induced Diabetic Peripheral Neuropathy

**Figure 1:**
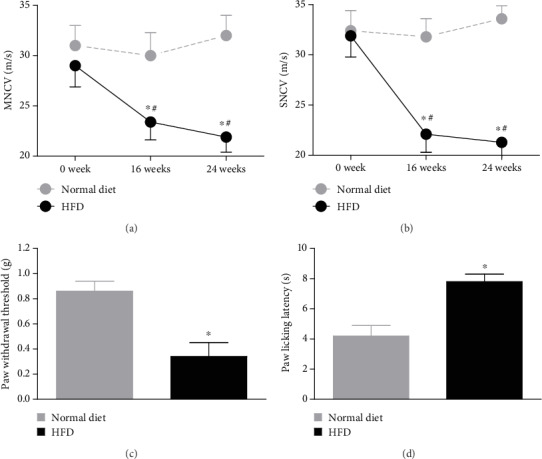
Effects of HFD treatment on neuropathy in mice. HFD-fed mice exhibited (a) motor nerve conduction velocity (MNCV), (b) sensory nerve conduction velocity (SNCV), (c) tactile allodynia, and (d) thermal hypoalgesia (*n* = 10/group). Asterisks represent the significant difference relative to normal diet control (*p* < 0.05). Pounds represent the significant difference relative to week 0 (*p* < 0.05).

Tactile allodynia and heat hypoalgesia tests were carried out in mice that received either normal diet or HFD for 24 weeks. Compared to normal diet mice, tactile allodynia and heat hypoalgesia were observed in HFD-fed mice (*t*-test, *p* < 0.01; Figure 1). We also measured the nerve conductive velocity and found that it was reduced in the HFD-fed mice (Figure 1).

### 3.3. Effects of HFD on Expression of A2ARs, Sema3a, Neuropilin 1, and Plexin A in the Spinal Cord

**Figure 2:**
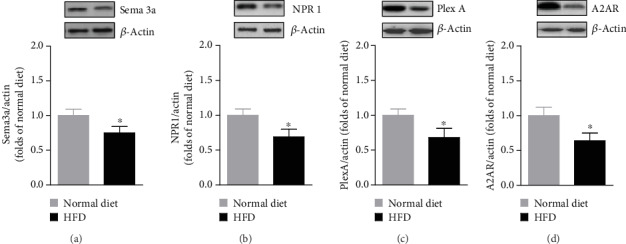
Effects of HFD treatment on Sema3a, NPR1, PlexA, and A2AR expression (*n* = 10/group). The spinal cords were collected at week 24. Western blot analysis was performed on (a) Sema3a, (b) NPR1, (c) PlexA, and (d) A2AR. Asterisks represent the significant difference relative to normal diet control (*p* < 0.05).

Compared to normal diet, HFD decreased the A2AR expression in the spinal cord at 24 weeks. HFD also decreased the expression of Sema3a in the spinal cord at 24 weeks relative to the normal diet (Figure 2(a)). In addition, the expression levels of Neuropilin 1 and Plexin A were also reduced after 24 weeks of HFD in comparison to normal diet (Figures 2(b) and 2(c)). Finally, the expression of A2AR in the spinal cord was also reduced after 24 weeks of HFD in comparison to the normal diet (Figure 2(d)).

### 3.4. Effects of IP Administrations of SCH58261 on HFD-Induced Peripheral Neuropathy

**Figure 3:**
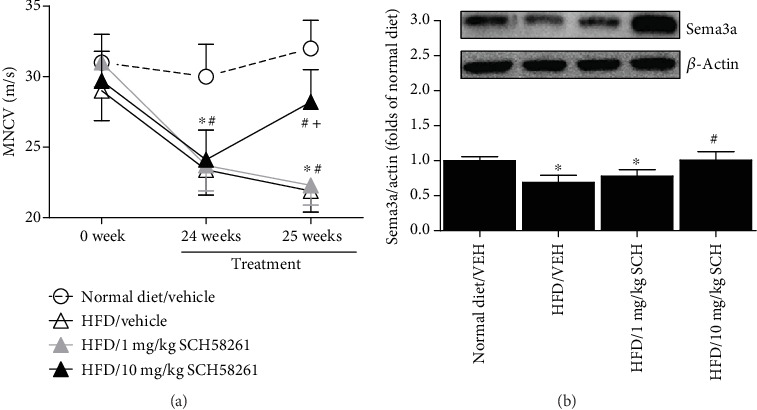
Effects of SCH58261 treatment on HFD-induced peripheral neuropathy in mice (*n* = 10/group). HFD-fed mice showed (a) slower motor nerve conduction velocity (MNCV) and (b) lower expression of Sema3a in the spinal cord. SCH58261 treatment (SCH) started at week 24 and was performed once daily for one week. The mice were tested for MNCV at week 25 and sacrificed for Western blot analysis. Asterisks represent the significant difference relative to normal diet control (*p* < 0.05). Pounds represent the significant difference relative to vehicle (VEH) (*p* < 0.05).

The mice receiving IP injections of vehicle showed slower nerve conduction velocity after 24 weeks of HFD (Figure 3(a)). However, the repeated once-daily injections of 10 mg/kg of SCH58261 (but not 1 mg/kg of SCH58261) have, in contrast to the vehicle, improved the nerve conduction velocity (Figure 3(a)). In addition, this phenomenon correlated with the enhanced Sema3a expression in the spinal cord of mice (Figure 3(b)).

### 3.5. Effects of IP Administrations of SCH58261 on IENFD

**Figure 4:**
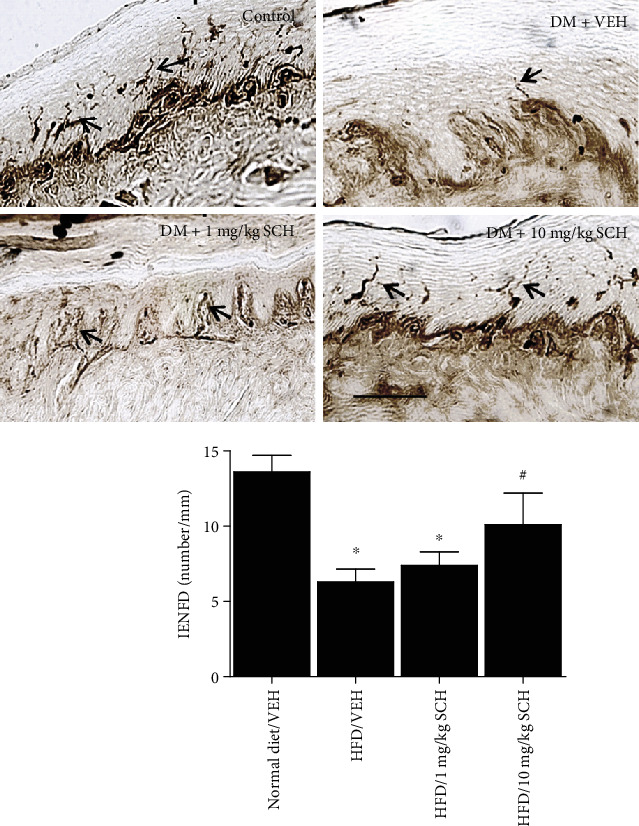
Effects of administration of SCH58261 on HFD-induced peripheral neuropathy in mice. Representative photomicrograph of intraepidermal nerve fiber profiles in control and diabetic mice treated with vehicle (VEH) or SCH58261 (SCH), magnification ×200. Arrows indicate the IENFs. Scale bar = 50 mm. Values are expressed as the means ± SEM, *n* = 10 per group. Asterisks represent the significant difference relative to normal diet control (*p* < 0.05). Pounds represent the significant difference relative to vehicle (*p* < 0.05).

Mice receiving IP injections of vehicle showed reduced IENFD after 24 weeks of HFD (Figure 4). However, the repeated once-daily injections of 10 mg/kg SCH58261 (but not 1 mg/kg of SCH58261) have, in contrast to the vehicle, improved IENFD (Figure 4).

## 4. Discussion

Our examination of A2AR in the HFD-induced peripheral neuropathy revealed that a time period of 24 weeks on HFD was long enough for the animals to develop tactile allodynia and thermal hypoalgesia. These observations are in agreement with the previously published data [26]. We made an interesting observation that the peripheral neuropathy caused by HFD was strongly associated with the decrease in the expression of Sema3a, Neuropilin 1, Plexin A, and A2AR in the spinal cord. Finally, the repeated administrations of selective A2AR agonist SCH58261 were found to attenuate these effects. These results clearly suggest a role of A2AR-mediated decrease of Sema3a signaling in the obesity-associated peripheral neuropathy.

It is well known that the majority of nerve repulsion factors, including Sema3a, and nerve elongation factors, including nerve growth factor, are produced by keratinocytes to regulate the epidermal innervation [30–34]. Our study reconfirmed that Sema3a is also expressed in the spinal cord. Diabetic peripheral neuropathy, which is characterized by a decrease in small fibers, is strongly connected with the hyperglycemic rate [35, 36]. Studies have shown that chronic hyperglycemia is associated with an increase in mRNA and protein expression of Sema3a in keratinocytes [37]. In contrast to these results, we observed the decreased Sema3a expression in the spinal cord of diabetic mice. While we are not clear about the reasons for this contradiction in Sema3a expression, our results point to the inhibitory effects of Sema3a on the neuropathic pain behaviors of diabetic mice. The connection between Sema3a and pain was discussed in several studies. In the model of corneal injury [38], Sema3a overexpression introduced through gene transfer suppressed the CGRP-positive nerve terminals and the mechanical stimuli hypersensitivity. The impaired AMPK-CGRP signaling in the central nervous system was shown to contribute to enhanced neuropathic pain in HFD-induced obese rats [39]. Furthermore, in the neuropathic pain model induced by NGF, the overexpression of the Sema3a by inoculating the Sema3a-expressing adenovirus induced the CGRP-positive tissue that sprouts in the dorsal horn [23]. There were, however, significant variations between these studies and our report in the used models of nerve injury and the interpretation of the experimental outcomes.

In various disorders, such as atopic dermatitis (AD), epidermal Sema3a may be strongly associated with IENFD [33, 34, 40]. Psoralen and ultraviolet A radiation therapies that were not reported to enhance the epidermal concentrations of Sema3a have also been reported to decrease IENFD in the skin of AD patients [33]. In addition, Sema3a replacement therapy normalizes AD hyperinnervation [40]. A recent study demonstrated that Sema3a levels are significantly increased in the suprabasal layer of the epidermis in both human patients and model animals with diabetic peripheral neuropathy [37]. Following the rapamycin treatment, the upregulation of Sema3a was concomitant with an elevation in the number of small fibers, without affecting hyperglycemia in diabetic rats. Other studies have found that Sema3a can inhibit DRG axon growth *in vitro* [31, 41]. Increased expression of Sema3a may lead to the collapse of the growth cone by linking to a Neuropilin 1/Plexin A receptor complex still expressed in small fibers of adult DRG cells [42]. These findings allow to hypothesize that increased Sema3a expression produced by keratinocytes might be associated with the decreased number of small fibers observed in diabetic peripheral neuropathy, and the decreased Sema3a expression in the spinal cord promotes the neuropathic pain often seen in diabetic patients.

## 5. Conclusions

Based on the results presented here, it is reasonable to assume that exposure to HFD can cause A2AR-regulated decrease in the spinal cord's Sema3a expression. Such regulation by A2AR seems to play an important role in the eventual development of peripheral neuropathy. Further, the dysregulated Sema3a signaling is relevant to the type 2 diabetes-associated peripheral neuropathy. The cases of the type 2 diabetes are on the rise, and a large proportion of population across the globe either suffers from type 2 diabetes or is prone to its development. In this context, it is of significance to further investigate the interactions and the interrelationships between A2AR and Sema3a. Deciphering of underlying mechanisms will lead to better understanding of the progression of diabetes and open up new avenues for therapeutic interventions, particularly in the context of complications such as peripheral neuropathy.

## Figures and Tables

**Table 1:** Body weight, one-day food intake, and blood glucose level in experimental animals.

Weeks post diet	Normal diet group	HFD group
1		
Body weight (g)	24.1 ± 0.4	28.8 ± 0.5^∗^
Average one-day food intake (g)	3.1 ± 0.5	4.6 ± 0.4^∗^
Blood glucose (mmol/l)	7.4 ± 0.5	7.5 ± 0.3
16		
Body weight (g)	36.3 ± 0.6	56.1 ± 1.2^∗^
Average one-day food intake (g)	3.1 ± 0.4	6.4 ± 0.4^∗^
Blood glucose (mmol/l)	7.4 ± 0.5	8.9 ± 0.5^∗^
24		
Body weight (g)	37.1 ± 2.1	57.4 ± 2.1^∗^
Average one-day food intake (g)	3.7 ± 0.4	6.9 ± 0.2^∗^
Blood glucose (mmol/l)	7.4 ± 0.6	9.0 ± 0.3^∗^

Data are expressed as means ± SEM, *n* = 9 − 10 per group. HFD: high-fat diet. Asterisks represent significant difference, relative to normal diet group. *P* < 0.05.

## Data Availability

The data used to support the findings of this study are available from the corresponding author upon request.
